# Leveraging high-resolution 7-tesla MRI to derive quantitative metrics for the trigeminal nerve and subnuclei of limbic structures in trigeminal neuralgia

**DOI:** 10.1186/s10194-021-01325-4

**Published:** 2021-09-23

**Authors:** Judy Alper, Alan C. Seifert, Gaurav Verma, Kuang-Han Huang, Yael Jacob, Ameen Al Qadi, John W. Rutland, Sheetal Patel, Joshua Bederson, Raj K. Shrivastava, Bradley N. Delman, Priti Balchandani

**Affiliations:** 1 BioMedical Engineering and Imaging Institute, Icahn School of Medicine at Mount Sinai, 1470 Madison Avenue; Floor 1, New York, NY 10029 USA; 2 Department of Biomedical Engineering, City College of New York, New York, NY USA; 3 Department of Neurosurgery, Mount Sinai Hospital, New York, NY USA; 4 Department of Diagnostic, Molecular, and Interventional Radiology, Icahn School of Medicine at Mount Sinai, New York, NY USA

**Keywords:** Trigeminal neuralgia, 7-tesla MRI, Thalamic subnuclei, Hippocampal subfields, Amygdala subnuclei, Pain

## Abstract

**Background:**

Trigeminal Neuralgia (TN) is a chronic neurological disease that is strongly associated with neurovascular compression (NVC) of the trigeminal nerve near its root entry zone. The trigeminal nerve at the site of NVC has been extensively studied but limbic structures that are potentially involved in TN have not been adequately characterized. Specifically, the hippocampus is a stress-sensitive region which may be structurally impacted by chronic TN pain. As the center of the emotion-related network, the amygdala is closely related to stress regulation and may be associated with TN pain as well. The thalamus, which is involved in the trigeminal sensory pathway and nociception, may play a role in pain processing of TN. The objective of this study was to assess structural alterations in the trigeminal nerve and subregions of the hippocampus, amygdala, and thalamus in TN patients using ultra-high field MRI and examine quantitative differences in these structures compared with healthy controls.

**Methods:**

Thirteen TN patients and 13 matched controls were scanned at 7-Tesla MRI with high resolution, T1-weighted imaging. Nerve cross sectional area (CSA) was measured and an automated algorithm was used to segment hippocampal, amygdaloid, and thalamic subregions. Nerve CSA and limbic structure subnuclei volumes were compared between TN patients and controls.

**Results:**

CSA of the posterior cisternal nerve on the symptomatic side was smaller in patients (3.75 mm^2^) compared with side-matched controls (5.77 mm^2^, *p* = 0.006). In TN patients, basal subnucleus amygdala volume (0.347 mm^3^) was reduced on the symptomatic side compared with controls (0.401 mm^3^, *p* = 0.025) and the paralaminar subnucleus volume (0.04 mm^3^) was also reduced on the symptomatic side compared with controls (0.05 mm^3^, *p* = 0.009). The central lateral thalamic subnucleus was larger in TN patients on both the symptomatic side (0.033 mm^3^) and asymptomatic side (0.035 mm^3^), compared with the corresponding sides in controls (0.025 mm^3^ on both sides, *p* = 0.048 and *p* = 0.003 respectively). The inferior and lateral pulvinar thalamic subnuclei were both reduced in TN patients on the symptomatic side (0.2 mm^3^ and 0.17 mm^3^ respectively) compared to controls (0.23 mm^3^, *p* = 0.04 and 0.18 mm^3^, *p* = 0.04 respectively). No significant findings were found in the hippocampal subfields analyzed.

**Conclusions:**

These findings, generated through a highly sensitive 7 T MRI protocol, provide compelling support for the theory that TN neurobiology is a complex amalgamation of local structural changes within the trigeminal nerve and structural alterations in subnuclei of limbic structures directly and indirectly involved in nociception and pain processing.

**Supplementary Information:**

The online version contains supplementary material available at 10.1186/s10194-021-01325-4.

## Introduction

Trigeminal neuralgia (TN) is a chronic and debilitating disorder, characterized by severe, sudden, and recurrent facial pain [1]. This condition affects 4.3 per 100,000 people in the United States every year [1] and despite existing pharmacological and surgical treatment options, longstanding efficacy remains relatively poor and potential complications and side effects are common [2].

Although TN clearly involves the trigeminal nerve (Cranial Nerve V, CNV), the precise etiology of TN remains obscure and is an ongoing topic of research [3, 4]. A wide range of etiologies have been described, including neurovascular compression (NVC) [5], multiple sclerosis [6], tumors [7], arteriovenous malformations [8], and facial injury [9] among others. NVC [1], as originally described by Janetta [10], is the most commonly accepted etiology for TN. The nerve is believed to be most susceptible to pathologic changes from mechanical compression in the root entry zone (REZ), or transition zone, just anterior to the nerve’s exit from the pons [9]. This region is thought to be most susceptible to vascular compression due to its proximity to normal blood vessels as well as fragility in the myelin associated with transition from oligodendrocyte to Schwann cell myelin. Vascular contact in the REZ can lead to demyelination and abnormal nerve conduction causing electrical instability, structural disarray, and atrophy [7].

While NVC is the most common observation in patients with TN, imaging studies suggest that TN can be present and recur in the absence of demonstrable NVC on MRI scans [11, 12]. In most cases, proof of compression is not required for treatment. Of those patients with confirmed NVC who are treated surgically, approximately 30% will experience recurrent pain within 10 years [13, 14]. Moreover, the appearance of NVC is often observed incidentally on MRI scans of individuals who do not suffer from TN [15]. The disparities between TN symptoms and NVC based on imaging, as well as the poor long-term efficacy of surgical treatment, suggest that NVC may not be the only cause of TN pain, or may cause changes in other parts of the nociceptive pathways that in turn cause TN symptomology in a large percentage of patients. Limited understanding of TN etiology is a significant impediment to developing effective treatments [1, 2, 16].

Multiple magnetic resonance imaging (MRI) studies have been performed to identify and characterize possible disease mechanisms of TN. It is becoming increasingly clear that while chronic pain in TN directly involves structural abnormalities at the level of compression at the trigeminal nerve REZ, with reduced cross-sectional area (CSA) and volume of the trigeminal nerve on the symptomatic side [17–20], broader structural disorder in pain-related limbic regions may be a critical component of TN pathophysiology as well [21, 22]. Tian et al. found differences in local functional connectivity density of the thalamus, hippocampus, and other regions outside the trigeminal nerve in TN [23]. Danyluk et al. showed that greater preoperative trigeminal nerve and hippocampal volumes may predict early non-response to surgical treatment for TN [24]. Zhang et al. demonstrated decreased grey matter volumes in the bilateral amygdala [22]. Vaculik et al. found volumetric reductions in ipsilateral hippocampal subfields in two cornu ammonis (CA) regions, CA1 and CA4, as well as in the dentate gyrus (DG) in TN patients [25]. Noorani et al. found that pain relief can reverse hippocampal abnormalities in trigeminal neuralgia and suggests that hippocampal volume reduction in TN is pain-driven [26]. These findings provide strong premise and motivation for further investigation of trigeminal nerve anatomy and subregions of limbic structures with hypothesized involvement in TN pain. The hippocampus, amygdala, and thalamus are of particular interest when it comes to exploring TN pathophysiology. The hippocampus is a stress-sensitive structure which can undergo changes in the presence of chronic pain [21, 25, 26]. The amygdala, which is the hub of the emotion-related network and involved in stress regulation, may be associated with pain in TN as well [21, 27]. Finally, the thalamus, which is involved in nociception and the trigeminal sensory pathway, is also implicated in TN [23, 28]. Further evaluation of these findings can promote a more comprehensive understanding of TN etiology and pain from localized nerve effects due to compression as well as more global, circuit-level effects.

Many of the MRI studies on TN have been performed at conventional clinical field strengths, including 1.5- and 3-Tesla, whose architecture and physics limit resolution and quantitative rigor especially for small structures. Clinical MRI protocols at conventional field strengths currently lack the sensitivity, specificity, and scope required to fully characterize elements of the TN pathway, such as trigeminal nerves themselves as well as subnuclei of limbic structures. Investigation of these subregions with increased anatomical detail can facilitate characterization of TN pathophysiology in these regions and may potentially identify novel targets for treatment and pain management.

Ultra-high field strength MRI scanners, including those operating at 7-Tesla (7 T), offer increased signal-to-noise ratio (SNR) [29] and enhanced contrast and resolution compared to conventional clinical scanners. 7 T imaging can reveal subtle anatomical alterations which are below the threshold of detectability at lower field strengths and enhance depiction of smaller structures including trigeminal nerves, hippocampal subfields, and subnuclei of the amygdala and thalamus. The purpose of this study is to leverage the enhanced SNR and resolution offered by ultra-high field MRI to quantify structural properties of the trigeminal nerve and calculate the volumes of subregions of the thalamus, hippocampus, and amygdala in patients with TN and matched healthy controls. This analysis, therefore, characterizes the primary and secondary brain regions that may be implicated in TN pathophysiology. We anticipate that the findings yielded through the highly sensitive imaging protocol in this study will aid in treatment options for TN as well as improve understanding of disease pathophysiology. Using structural neuroimaging to investigate changes in the nerve and limbic structure subnuclei in TN could provide a noninvasive method to monitor treatment progression, evaluate drug treatments in clinical trials, and potentially differentiate the different causes of TN based on underlying mechanisms.

## Methods

### Subjects

Patients with TN between the ages of 18 and 75 years old were recruited through our clinical collaborators (RS and JB) from the Department of Neurosurgery at Mount Sinai Hospital. All patients were diagnosed by a neurologist based on medical history and a physical examination. Subjects with a contraindication to MRI were excluded from the study. Written informed consent was provided by all subjects prior to the study, which was approved by the local Institutional Review Board. Clinical information, including disease duration, was obtained for all TN patients.

### Imaging protocol

Subjects underwent scanning with a Magnetom 7 T whole body MRI scanner (Siemens, Erlangen, Germany) equipped with 70 mT/m gradients (SC72CD, Siemens) and single-channel transmit, 32-channel receive head radiofrequency coil (Nova Medical, Wilmington, MA). The protocol included a 2D oblique-coronal T1-weighted MP2RAGE sequence for volumetric analysis, with the following parameters: TE 3.62 ms, TR 3000 ms, flip angle 7 degrees, matrix size 320 × 240, 224 slices, acceleration factor of 2, and 0.7 mm^3^ isotropic resolution.

### Nerve cross-sectional area

The cross-sectional area (CSA) of the nerve was measured by manually tracing regions of interest (ROIs) on high-resolution, oblique-coronal T_1_-weighted MP2RAGE images using Horos, an open source DICOM viewing software. Nerve ROIs were drawn by a trained image analyst (JA) and guided by a neuroradiologist (BD) with Certificate of Added Qualification (CAQ) in neuroradiology. A total of four ROIs were traced for each subject, with two ROIs traced on the left trigeminal nerve and two on the right trigeminal nerve. For each nerve, the first ROI was traced at the most posterior point where the nerve emerges from the pons and the second ROI at the most anterior point just before the nerve enters Meckel’s cave. The CSA was quantified for each nerve ROI and group comparisons of CSA values on the symptomatic and asymptomatic sides were performed.

### Segmentation of subnuclei of limbic structures

Segmentation of the hippocampus, amygdala, and their subfields were performed using an automated segmentation software, Freesurfer image analysis suite version 6.0 (http://surfer.nmr.mgh.harvard.edu). Segmentation of the thalamus and its subnuclei was performed using Freesurfer version 7.0 [ 30]. A summary of the regions analyzed is shown in Fig. 1 (see Additional file 2).

### Statistical analysis

All limbic structure subnuclei volumes were normalized to total brain volume, which was also provided by Freesurfer 6.0. We performed two-tailed, independent Student’s *t*-tests to compare structural data between TN patients and controls, with the symptomatic side in patients compared to the same side in matched healthy controls (side-matched). *P* < 0.05 was considered significant. Effect sizes (*Cohen’s d*) were also calculated for each comparison.

Due to the heterogeneity in the trigeminal neuralgia patients, analyses were also performed on the subset of nine classical, purely paroxysmal trigeminal neuralgia patients for all significant findings in the larger group.

A total of two nerve ROIs, six hippocampal regions, ten amygdala regions, and twenty-six thalamic regions were quantified for each nerve and compared between patients and controls. Correction for multiple comparisons was performed with false discovery rate (FDR) correction.

Since our *t*-tests assume a standard distribution, we performed permutation testing, a type of statistical significance test, to check the significant values for false positives and verify the statistical significance of our model. The method of permutation testing is similar to that performed in a study by Jacob et al. [31] With permutation testing, we obtained the distribution of the test statistic under the null hypothesis, which would be no significant differences between the patient and healthy control groups. This involved combining and then shuffling the sample patient and control values randomly one thousand times (k = 1000) to create a simulated population distribution of the test statistic for each random rearrangement of our data points. We then compared it to our data’s t-statistic to obtain a permutation-derived *p* value. This is especially beneficial for our study containing multiple statistical tests, since the assumptions and/or approximations required for parametric tests may not be satisfied for each and every test and correlation across tests can be difficult to account for. While it is not a correction for multiple comparisons, permutation testing is beneficial as a method to determine whether the significant findings are due to random chance or driven by real effects.

We also investigated the correlation of structural changes in TN with disease duration for each significant subnucleus finding. The correlations were performed using linear regression.

## Results

Thirteen TN patients (mean age 42.9 years, standard deviation 12.2 years, 5 males and 8 females) and thirteen healthy controls matched for age and gender (mean age 42.5 years, standard deviation 10.2 years) were included in this study. Age was not significantly different between groups (*p* = 0.9). There were 23% more females than males in the cohort, which reflects the greater female prevalence of the disease in the general population [1]. Nine patients had classical, purely paroxysmal trigeminal neuralgia and the remaining four patients had secondary trigeminal neuralgia, as determined by ICHD-3 criteria. Clinical information for all TN patients are provided in Table 1 (see Additional file 1).

### Structural changes in TN patients

Table 2 (see Additional file 1) includes a summary of significant findings (defined as *p* < 0.05) and their effect sizes in the full sample of TN patients compared to controls. The significant findings did not survive FDR correction for multiple comparisons. However, permutation testing performed on each of these findings resulted in significant permutation-derived *p* values (defined as *p* < 0.05) and are included in the table as well.

#### Nerve cross sectional area

The CSA of the trigeminal nerve at exit from the pons was found to be significantly lower in TN patients on the symptomatic side (3.75 mm^2^, *p* = 0.0057, *d* = − 1.19) compared to the same side in healthy controls (5.77 mm^2^). No significant difference in CSA was found on the asymptomatic side.

Differences in CSA of the anterior point along the nerve where it enters Meckel’s cave did not reach significance on either the symptomatic or asymptomatic side.

#### Hippocampal subfield volumes

Volumetric differences were evaluated in segmented hippocampal subfields of interest, including CA1, CA3, CA4, DG, subiculum, and the whole hippocampus on each side. When comparing patients to controls, the volumetric differences in these regions did not reach significance on either the symptomatic or asymptomatic side.

#### Amygdala subnuclei volumes

The basal nucleus of the amygdala was found to be significantly smaller on the symptomatic side in patients (0.35 mm^3,^ *p* = 0.025, *d* = − 0.94) compared to the corresponding side in controls (0.40 mm^3^). No significant difference in basal nucleus volume was found on the asymptomatic side. The paralaminar nucleus was also found to be significantly smaller in patients (0.039 mm^3^*, p* = 0.009, *d* = − 1.12) compared to controls (0.047 mm^3^) on the symptomatic side. The other amygdala subnuclei segmented, including lateral, accessory-basal, anterior-amygdaloid, central, medial, cortical, and cortico-amygdaloid, as well as total amygdala volume, were not significantly different between patients and controls on the symptomatic or asymptomatic side.

#### Thalamic subnuclei volumes

The central lateral (CL) subnucleus ipsilateral to TN was significantly larger in patients (0.033 mm^3^, *p* = 0.048, *d* = 0.819) compared to controls (0.025 mm^3^). The CL subnucleus on the side contralateral to pain was also found to be larger in patients (0.035 mm^3^, *p* = 0.003, *d* = 1.32) compared to controls (0.025 mm^3^). The lateral pulvinar (PuL) subnucleus on the symptomatic side was found to be significantly smaller in patients (0.17 mm^3^, *p* = 0.042, *d* = − 0.84) compared to controls (0.18 mm^3^). The inferior pulvinar (PuI) subnucleus on the symptomatic side was also found to be significantly smaller in patients (0.1998 mm^3^, *p* = 0.044, *d* = − 0.83) compared to controls (0.226 mm^3^). Other thalamic subnuclei in the anterior, lateral, ventral, intralaminar, medial, and posterior, as well as total thalamic volume, were not significantly different between patients and controls on either the symptomatic or asymptomatic side.

Illustrative ROIs and segmentation results in a single TN subject, which provide the basis of the CSA and volumetric analyses, are shown in Fig. 2 (see Additional file 3).

### Structural changes in classical, purely paroxysmal TN patients

Due to the heterogeneity in the trigeminal neuralgia patients, analyses were performed on the subset of nine classical, purely paroxysmal TN patients for all significant findings in the larger group. Table 3 (see Additional file 1) includes a summary of significant findings in the subset of nine classical, purely paroxysmal TN patients, and their effect sizes. Permutation testing performed on each of these findings resulted in significant permutation-derived *p* values (defined as *p* < 0.05) and are included in the table.

#### Nerve cross sectional area in classical subset

The CSA of the trigeminal nerve at the point of exit from the pons was found to be significantly lower in classical, purely paroxysmal TN patients on the symptomatic side (3.12 mm^2^, *p* = 0.0075, *d* = − 1.44) compared to the same side in healthy controls (5.67 mm^2^).

#### Amygdala subnuclei volumes in classical subset

The basal nucleus of the amygdala was found to be significantly smaller on the symptomatic side in classical, purely paroxysmal patients (0.33 mm^3^, *p* = 0.017, *d* = − 1.26) compared to the corresponding side in controls (0.41 mm^3^). However, the paralaminar nucleus on the symptomatic side, was not found to be significantly smaller in this subset of patients compared to controls.

#### Thalamic subnuclei volumes in classical subset

The central lateral (CL) subnucleus on the side contralateral to pain was found to be larger in this subset of classical, purely paroxysmal patients (0.037 mm^3^, *p* = 0.0074, *d* = 1.45) compared to controls (0.030 mm^3^). The inferior pulvinar (PuI) subnucleus on the symptomatic side was found to be significantly smaller in these patients (0.20 mm^3^, *p* = 0.0052, *d* = − 1.52) compared to controls (0.23 mm^3^). However, the finding in the lateral pulvinar (PuL) on the symptomatic side did not persist in this TN subset. The finding in the CL subnucleus ipsilateral to TN also did not persist in this TN subset and had only borderline significance with a large effect size (*p* = 0.051, *d* = 0.99).

### Relationship between structural changes and disease duration

Linear regressions were performed for each significant subnucleus volume difference versus disease duration. No statistically significant correlations were found in the full TN cohort, nor in the subset of classical, purely paroxysmal patients.

## Discussion

Advanced imaging studies are increasingly being applied to study TN. Studies have shown structural changes in trigeminal nerves [17–20] and limbic structures [21, 23–26] in the presence of TN pathology. In this study, ultra-high field MRI provided excellent resolution of the trigeminal nerves as well as granular subregions of the hippocampus, amygdala, and thalamus. The purpose of this exploratory study was to identify potential structural changes which may be associated with TN pathophysiology and that can be further explored in future studies. Analyses including comparison of CSA of the nerves and volumes of limbic structure subregions between patients and controls were performed.

### Structural changes in TN patients

The data in this pilot study revealed that the CSA of the trigeminal nerve was reduced on the symptomatic side, specifically in the region where the nerve exits the pons, in patients compared to controls. The symptomatic side corresponded to the compressed side for patients with NVC. Although the exact cause of reduced CSA, such as compression or pathologic volume loss, is not identifiable given our data, reduced CSA of the nerve on the symptomatic side in patients compared to controls is consistent with the hypothesis that NVC plays a role in TN. Reduced CSA was shown at the posterior point along the nerve’s trajectory where it exits the brainstem, and not where the nerve enters Meckel’s cave. This is consistent with the theory that the compression of posterior portion of the nerve is more likely to be associated with symptomatic TN than compression of the anterior portion.

The data also showed differences between patients and controls in subnuclei of limbic structures with hypothesized involvement in TN, including the basal and paralaminar subnuclei in the amygdala on the symptomatic side, the inferior and lateral pulvinar thalamic subnuclei on the symptomatic side, and the central lateral nucleus of the thalamus on the symptomatic and asymptomatic sides.

The basal nucleus is one of the main sources of amygdala output to the ventral striatum which is involved in modulating pain [32]. We observed reduced basal nucleus volume in TN patients, potentially indicating inadequate pain modulation and processing in TN. The function of the paralaminar subnucleus of the amygdala is poorly understood at this time [33]. We observed reduced paralaminar nucleus volume in TN suggesting that this region may play a role in pain.

The thalamic subnuclei volumes which appear significantly different between patients and controls in our analysis do not necessarily represent the areas expected to be involved in TN neurobiology. While the thalamic subnucleus most directly involved in nociception in the trigeminal sensory pathway is the contralateral ventral posteromedial nucleus (VPM), no significant volume loss was noted in our analysis. Instead, we observed increased volume in the central lateral nucleus of the thalamus in TN patients on the symptomatic side in the full TN patient cohort and the asymptomatic side in both the full cohort and the subset of classical, purely paroxysmal TN patients. Multiple studies have established the posterior part of the CL (CLp) nucleus as a target in chronic therapy-resistant neurogenic pain, including trigeminal pain. The CLp transfers nociceptive information through the spinothalamic and spinoreticulothalamic pathways to large areas of cortex, including the insula and anterior cingulate cortex which are involved in nociception [34]. Although trigeminal sensation is carried in the trigeminothalamic tract which is distinct from the spinothalamic tract, they are analogous in that they are both involved in nociception. The increased volume of the CL subnucleus observed on both the symptomatic and asymptomatic sides in TN patients may indicate a systemic, compensatory effect in response to neurogenic pain.

While the pulvinar subnucleus of the thalamus is thought to be mainly involved in processing visual stimuli, this region is proposed to also be involved in proprioception and multisensory processing, including pain [35]. This supports the notion that the reduced inferior and lateral pulvinar volumes we observed on the symptomatic side in TN patients may reflect the role of the pulvinar in pain processing.

Additional analyses, including t-tests and permutation tests, were performed on the homogenous subgroup for all significant findings in the full patient cohort. Although the sample size of this subset is < 10, it was important to perform further analysis to avoid mixing TN diseases with diverse clinical manifestation and pathophysiology. Since there were significant findings which persisted in this homogenous subset, especially in the nerve CSA, we were able to determine that these findings were not due to tumor-related compressive effects.

### Limitations and future directions

One limitation of this study is the small sample size. This was an exploratory study in a rare disease, limiting the rate of recruitment. Despite this, potentially significant differences were found between TN patients and healthy controls in this dataset, providing targeted nerve and limbic subnuclei regions for future high resolution 7 T studies. Analysis with a larger sample size is needed to validate these findings with sufficient power.

The source of CSA reduction is unknown; potential causes could be compression or atrophy. Ascertaining the mechanism of CSA reduction is beyond the scope of this study and may be investigated using a study with a larger sample size containing patients with several TN etiologies. It is possible that compression leads to nerve atrophy over time, but since the patients in this study were imaged prior to intervention it is also possible that the finding of reduced CSA represents the compression itself rather than atrophy.

The findings reported in this study in areas beyond the confines of the trigeminal nerves (i.e., the amygdala and thalamus) may constitute downstream effects and abnormalities that might potentiate, contribute or enhance TN. Effects in brain regions related to chronic pain, specifically in granular limbic structure regions with potential involvement in pain modulation and processing, require further study with a larger dataset to be fully characterized.

While the subjects in this study reflected the greater prevalence of TN in females compared to males, a comparison of structural findings in the two sexes was not feasible in our cohort, because of the small sample size of the male and female subsets. This analysis would be warranted in a future study with a larger cohort.

Prior literature suggests that hippocampal changes may differ in males versus females as hormones have been shown to play a role in hippocampal abnormalities in animal [36–38] and human studies [39–41]. A larger study is warranted to evaluate correlations between changes in hippocampal subfield volumes and sex in a TN cohort as future work. Since hippocampal volume is also sensitive to baseline cortisol levels [42], this study would need to control for individual cortisol levels in the cohort.

It has also been demonstrated that some drugs used for TN, such as antiepileptic drugs, can result in morphological neurodegenerative changes in animal models [43] as well as reduced hippocampal betweenness centrality (which is a measure of functional connectivity) in humans [44]. Evaluation of drug effects on neurodegenerative imaging biomarkers is warranted in future studies with a larger sample size.

Although no effects were shown in subnucleus volumes versus disease duration, this correlation should be explored further in future work with a larger cohort to validate previous findings which suggest that duration of symptoms may be linked to structural alterations in TN^26^. Future work evaluating the intensity of TN attacks, presence of sensory deficit, and allodynia can further elucidate the relationship between pain and structural changes in TN.

## Conclusions

This study is a preliminary investigation of structural alterations in the trigeminal nerve and subnuclei of limbic structures in TN patients using ultra-high field MRI and examines quantitative differences in these structures versus healthy controls. These results provide compelling support for the theory that TN pathophysiology is a complex mixture of local structural alterations in the trigeminal nerve as well as alterations in the structure and, potentially, function of various brain regions directly and indirectly involved in nociception and pain processing.

## Supplementary Information

**Additional file 1: Table S1.** Demographics and disease properties of TN patients in this study. (CN V = trigeminal nerve, Lt = left, Rt = right, REZ = root entry zone). **Table S2.** Significant results for comparison of trigeminal neuralgia patients to healthy controls. **Table S3.** Significant results for comparison of classical, purely paroxysmal trigeminal neuralgia patients to healthy controls.

**Additional file 2: Figure S1.** Depiction of left hemisphere regions and subregions analyzed in this study including trigeminal nerve cross-sectional area anteriorly and posteriorly, hippocampal subfield volumes, amygdala subnuclei volumes, and thalamic subnuclei volumes. The thalamus is shown as an axial slice rotated 90 degrees on its horizontal axis. (CA = cornu ammonis, DG = dentate gyrus, ROI = region of interest).

**Additional file 3: Figure S2.** Results displayed on 7 T T1-weighted images for one TN patient. Panel A: Delineation of right and left posterior ROIs drawn on a coronal slice. Panel B: Segmentation of hippocampal subfields on a coronal slice. Panel C: Segmentation of amygdala subnuclei on a coronal slice. Panel D: Segmentation of thalamic subnuclei on an axial slice. (CA = cornu ammonis, GC-DG = granule cell layer of dentate gyrus, PuL = lateral pulvinar, PuM = medial pulvinar, VA = ventral anterior, VLa = ventral lateral anterior, VLp = ventral lateral posterior, VPL = ventral posterolateral, MDl = mediodorsal lateral parvocellular, MDm = mediodorsal medial magnocellular, PuA = anterior pulvinar, AV = anteroventral, CL = central lateral).

## Data Availability

The dataset supporting the conclusions of this article is included within the article and its additional files.

## Abbreviations

TN: Trigeminal neuralgia
NVC: Neurovascular compression
CSA: Cross sectional area
CNV: Cranial nerve V
REZ: Root entry zone
MRI: Magnetic resonance Imaging
CA: Cornu ammonis
DG: Dentate gyrus
7 T: 7-Tesla
SNR: Signal-to-noise ratio
RS: Raj Shrivastava
JB: Joshua Bederson
ROIs: Regions of interest
JA: Judy Alper
BD: Bradley Delman
CAQ: Certificate of added qualification
FDR: False discovery rate
CL: Central lateral
PuL: Lateral pulvinar
PuI: Inferior pulvinar
VPM: Ventral posteromedial
CLp: Posterior central lateral
