# Supplementary material for: Leveraging high-resolution 7-tesla MRI to derive quantitative metrics for the trigeminal nerve and subnuclei of limbic structures in trigeminal neuralgia
Source: J Headache Pain. 2021 Sep 23;22(1):112. doi: 10.1186/s10194-021-01325-4 (PMC8461944; doi:10.1186/s10194-021-01325-4)
Supplement: Supplementary file 1 — Additional file 1: Table S1. Demographics and disease properties of TN patients in this study. (CN V = trigeminal nerve, Lt = left, Rt = right, REZ = root entry zone). Table S2. Significant results for comparison of trigeminal neuralgia patients to healthy controls. Table S3. Significant results for comparison of classical, purely paroxysmal trigeminal neuralgia patients to healthy controls. [file 10194_2021_1325_MOESM1_ESM.doc]

Table 1. Demographics and disease properties of TN patients in this study. (CN V= trigeminal nerve, Lt = left, Rt = right, REZ= root entry zone)

| **Patient Number** | **Sex** | **Age (years)** | **Age at Onset (years)** | **Disease Duration (Months)** | **Symptomatic Side** | **NVC in Surgery** | **Classification** | **Distribution** | **Imaging Features from Clinical and 7T MRI Radiological Reads** | **Treatment at time of Study** |
| --- | --- | --- | --- | --- | --- | --- | --- | --- | --- | --- |
| 1 | M | 45 | 41 | 48 | Left | artery and vein | Classical, Purely Paroxysmal | V2 | Compression of Lt CN V through a vascular loop | Trigeminal injections, baclofen |
| 2 | F | 66 | 56 | 120 | Right | vein | Classical, Purely Paroxysmal | V2, V3 | Vascular compression in close proximity to Rt REZ, 1 mm enhancement in Rt Meckel's cave, which may indicate dilated vessel loop | Gabapentin, Trileptal, baclofen |
| 3 | F | 48 | 46 | 24 | Left | tumor-epidermoid | Secondary | V2, V3 | Lt posterior fossa epidermoid lesion with extension into prepontine cistern and quad-trigeminal plate, clear compression and elevation of CN V at brainstem origin | Neuropathic pain medications |
| 4 | F | 27 | 27 | 12 | Left | tumor-epidermoid | Secondary | V2 | Lt cerebellopontine angle epidermoid lesion extending from internal auditory canal to the back of the clivus and Dorello's canal, causing compression of CN V | None |
| 5 | M | 42 | 31 | 144 | Right | artery | Classical, Purely Paroxysmal | V2 | Compressive vessel around brainstem interface of the Rt CN V | Neuropathic pain medications |
| 6 | F | 61 | 58 | 36 | Right | artery | Classical, Purely Paroxysmal | V2, V3 | Vascular compression at the Rt brainstem origin of CN V | Oxcarbazepine.  baclofen |
| 7 | F | 44 | 43 | 12 | Right | NA (no surgery) | Classical, Purely Paroxysmal | V2, V3 | Vascular compression of Rt trigeminal complex | Baclofen |
| 8 | F | 38 | 36 | 2 | Right | tumor | Secondary | V1 | Large Rt greater sphenoid wing mass (meningioma) with extension and obliteration into Rt cavernous sinus; tumor effacement and nonvisualization of Rt trigeminal ganglion except for Meckel's sparing posteriorly | Gabapentin |
| 9 | M | 46 | 46 | 5 | Right | NA (no surgery) | Classical, Purely Paroxysmal | V2, V3 | No clear NVC, superior cerebellar tributaries run in the vicinity of the upper Rt trigeminal REZ | Trilpetal, neurontin |
| 10 | M | 31 | 31 | 4 | Left | NA (no surgery) | Classical, Purely Paroxysmal | V1, V2 | Compression lesion via the Lt REZ | Neuropathic pain medications |
| 11 | F | 49 | 44 | 60 | Right | artery | Secondary | V1, V2, V3 | Duplicated Rt superior cerebellar artery with NVC of the Rt trigeminal REZ | Neuropathic pain medications |
| 12 | F | 23 | 23 | 18 | Right | artery and vein | Classical, Purely Paroxysmal | V1, V2 | Vascular compression at Rt CN V root origin | Oxcarbozine.  gabapentin |
| 13 | M | 38 | 37 | 12 | Right | artery | Classical, Purely Paroxysmal | V2 | Ascending artery conflict with Rt CN V REZ | Tegretol |

Table 2. Significant results for comparison of trigeminal neuralgia patients to healthy controls.

| **Region** | **Subregion** | **Property** | **Symptomatic Side** | **Patient mean**  **(n=13)** | **Control mean**  **(n=13)** | ***p* value (t-test)** | **Cohen's *d*** | ***p* value (permutation)** |
| --- | --- | --- | --- | --- | --- | --- | --- | --- |
| **Trigeminal Nerve** | Posterior Root Entry Zone | CSA (mm^2^) | Ipsilateral | 3.75 | 5.77 | 0.0057 | -1.19 | 0.0080 |
| **Amygdala** | Basal nucleus | Volume (mm^3^) | Ipsilateral | 0.35 | 0.40 | 0.025 | -0.94 | 0.0010 |
|  | Paralaminar nucleus | Volume (mm^3^) | Ipsilateral | 0.040 | 0.047 | 0.0086 | -1.12 | 0.0020 |
| **Thalamus** | Pulvinar inferior | Volume (mm^3^) | Ipsilateral | 0.20 | 0.23 | 0.044 | -0.83 | 0.020 |
|  | Central lateral | Volume (mm^3^) | Ipsilateral | 0.033 | 0.025 | 0.048 | 0.82 | 0.024 |
|  |  | Volume (mm^3^) | Contralateral | 0.035 | 0.025 | 0.0025 | 1.32 | 0.0010 |
|  | Pulvinar lateral | Volume (mm^3^) | Ipsilateral | 0.17 | 0.18 | 0.042 | -0.84 | 0.036 |

Table 3. Significant results for comparison of classical, purely paroxysmal trigeminal neuralgia patients to healthy controls.

| **Region** | **Subregion** | **Property** | **Symptomatic Side** | **Patient mean**  **(n=9)** | **Control mean**  **(n=9)** | ***p* value (t-test)** | **Cohen's *d*** | ***p* value (permutation)** |
| --- | --- | --- | --- | --- | --- | --- | --- | --- |
| **Trigeminal Nerve** | Posterior Root Entry Zone | CSA (mm^2^) | Ipsilateral | 3.12 | 5.67 | 0.0075 | -1.44 | 0.0060 |
| **Amygdala** | Basal nucleus | Volume (mm^3^) | Ipsilateral | 0.33 | 0.41 | 0.017 | -1.26 | 0.0070 |
| **Thalamus** | Pulvinar inferior | Volume (mm^3^) | Ipsilateral | 0.20 | 0.23 | 0.0052 | -1.52 | 0.0060 |
|  | Central lateral | Volume (mm^3^) | Contralateral | 0.037 | 0.030 | 0.0074 | 1.45 | 0.045 |
