# Supplementary figures and images for: Leveraging high-resolution 7-tesla MRI to derive quantitative metrics for the trigeminal nerve and subnuclei of limbic structures in trigeminal neuralgia
Source: J Headache Pain. 2021 Sep 23;22(1):112. doi: 10.1186/s10194-021-01325-4 (PMC8461944; doi:10.1186/s10194-021-01325-4)

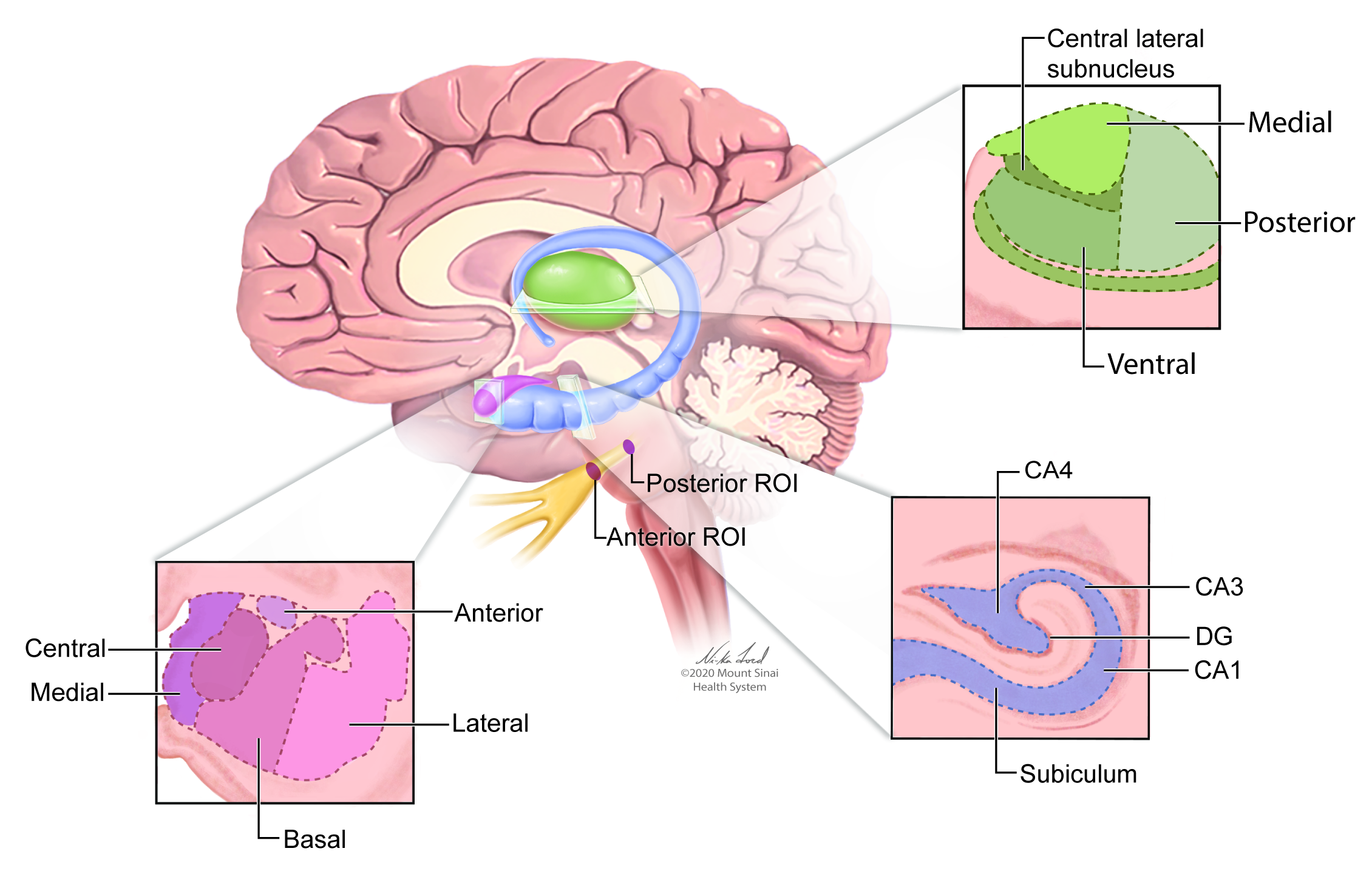

Supplement: Supplementary file 2 — Additional file 2: Figure S1. Depiction of left hemisphere regions and subregions analyzed in this study including trigeminal nerve cross-sectional area anteriorly and posteriorly, hippocampal subfield volumes, amygdala subnuclei volumes, and thalamic subnuclei volumes. The thalamus is shown as an axial slice rotated 90 degrees on its horizontal axis. (CA = cornu ammonis, DG = dentate gyrus, ROI = region of interest). [file 10194_2021_1325_MOESM2_ESM.tif]

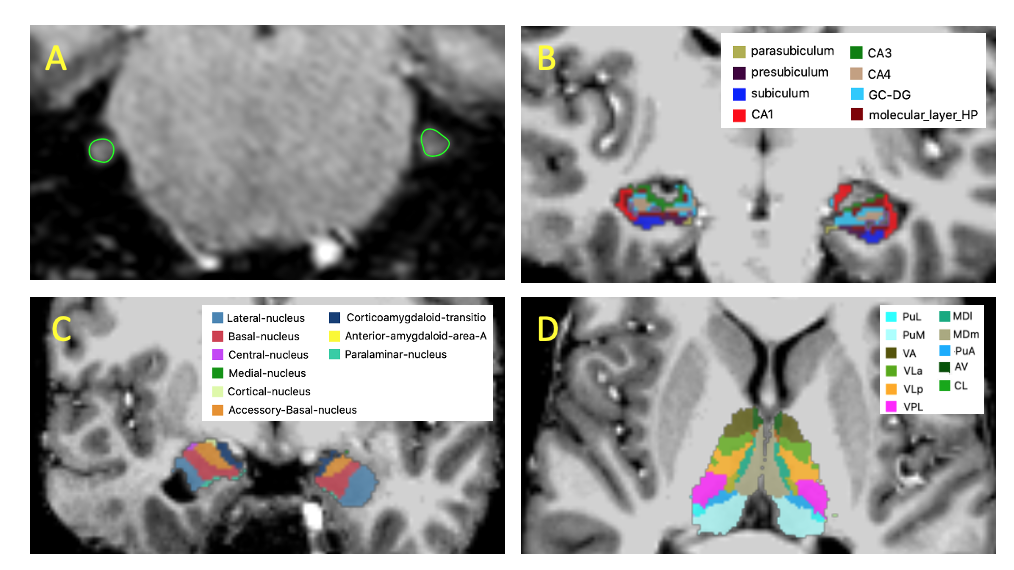

Supplement: Supplementary file 3 — Additional file 3: Figure S2. Results displayed on 7 T T1-weighted images for one TN patient. Panel A: Delineation of right and left posterior ROIs drawn on a coronal slice. Panel B: Segmentation of hippocampal subfields on a coronal slice. Panel C: Segmentation of amygdala subnuclei on a coronal slice. Panel D: Segmentation of thalamic subnuclei on an axial slice. (CA = cornu ammonis, GC-DG = granule cell layer of dentate gyrus, PuL = lateral pulvinar, PuM = medial pulvinar, VA = ventral anterior, VLa = ventral lateral anterior, VLp = ventral lateral posterior, VPL = ventral posterolateral, MDl = mediodorsal lateral parvocellular, MDm = mediodorsal medial magnocellular, PuA = anterior pulvinar, AV = anteroventral, CL = central lateral). [file 10194_2021_1325_MOESM3_ESM.tiff]
